# Soybean Interaction with Engineered Nanomaterials: A Literature Review of Recent Data

**DOI:** 10.3390/nano9091248

**Published:** 2019-09-03

**Authors:** Vasile Coman, Ioana Oprea, Loredana Florina Leopold, Dan Cristian Vodnar, Cristina Coman

**Affiliations:** 1 Institute of Life Sciences, University of Agricultural Sciences and Veterinary Medicine, 400372 Cluj-Napoca, Romania (V.C.) (I.O.) (L.F.L.) (D.C.V.); 2 Faculty of Food Science and Technology, University of Agricultural Sciences and Veterinary Medicine, 400372 Cluj-Napoca, Romania

**Keywords:** engineered nanomaterials, phytotoxicity, plant microbiota, soybean

## Abstract

With a continuous increase in the production and use in everyday life applications of engineered nanomaterials, concerns have appeared in the past decades related to their possible environmental toxicity and impact on edible plants (and therefore, upon human health). Soybean is one of the most commercially-important crop plants, and a perfect model for nanomaterials accumulation studies, due to its high biomass production and ease of cultivation. In this review, we aim to summarize the most recent research data concerning the impact of engineered nanomaterials on the soya bean, covering both inorganic (metal and metal-oxide nanoparticles) and organic (carbon-based) nanomaterials. The interactions between soybean plants and engineered nanomaterials are discussed in terms of positive and negative impacts on growth and production, metabolism and influences on the root-associated microbiota. Current data clearly suggests that under specific conditions, nanomaterials can negatively influence the development and metabolism of soybean plants. Moreover, in some cases, a possible risk of trophic transfer and transgenerational impact of engineered nanomaterials are suggested. Therefore, comprehensive risk-assessment studies should be carried out prior to any mass productions of potentially hazardous materials.

## 1. Introduction

Nanotechnology, together with molecular biology and information technology, has brought new insights into the understanding of our life on Earth [1]. Products of nanotechnology, such as engineered nanomaterials (ENMs), are one of the new and exciting discoveries of the past century. ENMs, which are materials with dimensions in the range where biological interactions are imminent, have contributed to many revolutionary developments, ranging from energy production and storage, agricultural and environmental applications, and up to targeted drug delivery systems [2,3,4,5,6,7,8].

Unfortunately, extended manufacturing and use generally cause a significant amount of ENMs to be released into the environment (air, water, landfills and soil) [1,9]. ENM properties are most often different from those of bulk materials, sometimes posing environmental and health risks. In time, ENMs accumulate, especially in soils, and may become hazardous to soil bacteria and edible plants [10]. Plant biomass accounts for approximately 80% of the total biomass on Earth [11]. Therefore, plants can have a crucial role in the circulation of ENMs in ecosystems, their biotransformation, and bioaccumulation in the food chain [12]. ENMs can be potentially taken up from the soil by plants via their roots, and transported to their shoots, leaves and seeds, depending upon ENM size, shape, surface characteristics and charge [13,14].

Soybean (*Glycine max*), one of the most commercially-important crop plants in terms of seed protein and oil contents, has proven to be a perfect model for ENM accumulation studies due to its high biomass production and ease of cultivation. Moreover, the sequencing of its full genome has opened new possibilities in terms of the crop improvements needed for human and animal food production [15]. In this context, it is important in addition to evaluate the abiotic factors which may interact with the growth and yield of this crop. In the past two decades, due to important advancements in analytical and imaging techniques, numerous studies have aimed to clarify and quantify the interactions between ENMs and edible plants. However, the full toxicological profile of ENMs is far from being completely evaluated.

In this review, we aim to put into a general context (plant interactions with ENMs) the most recent research data evaluating the impact of ENMs on soybean plants, in terms of positive and negative effects on growth, production, metabolism and interaction with the plant-associated microbiota. In terms of ENMs, we aim to summarize recent studies investigating both inorganic (metal and metal-oxide nanoparticles (NPs)), and organic (carbon-based) ENMs. Finding the optimal conditions where ENM exposure is non-toxic, or even beneficial to plants, can play an important role in a future sustainable quality food production.

## 2. Engineered Nanomaterials and the Environment

There is no generally agreed definition of nanomaterials (NMs). The European Union (EU) adopted a definition of a NM through the directive 2011/696/EU, by which a NM is “a natural, incidental or manufactured material containing particles, in an unbound state or as an aggregate or as an agglomerate and where, for 50 % or more of the particles in the number size distribution, one or more external dimensions is in the size range of 1 nm to 100 nm.” Thus, nanofilms (one nm dimension), nanowires, and nanotubes (two nm dimensions), as well as nanoparticles (NPs) (three nm dimensions) fall within the NM definition.

As very relevantly and comprehensively summarized in a relatively recent review [1], there are three main types of NMs: (i) Natural—found in the bodies of living organisms; (ii) incidental—usually, byproducts of natural and industrial phenomena; and (iii) engineered (ENMs)—produced by humans, with desired properties for certain applications. Most ENMs are less than a century old, and represent a very small fraction compared to the natural and incidental NMs [1]. ENMs can be classified into organic NMs (carbon nanotubes, graphenes and fullerenes), inorganic NMs (metals and metal oxides), organometallic NMs (modified metallic surfaces and polymer–nanoclay composites), and semiconductor nanocrystals (quantum dots) in the size range 1–100 nm, with chemical, physical and electrical properties that change as a function of the size and shape of the material and the surface functionalization [1,16]. The most abundant ENMs are metal and metal oxides (e.g., TiO_2_, SiO_2_, Fe, ZnO, Al_2_O_3_, CeO_2_, Cu, and Ag), carbon nanotubes and graphenes, and nanoclay composites [9].

The unique general properties of NMs (particle size, shape, charge, surface area and reactivity) compared to their bulk or dissolved counterparts, promoted the production of key products containing NMs in the energy [17] and electronics [4], agriculture [7,18] and water treatment [8], personal care and biomedical sectors [2,3,5]. In 2014, there were about 1,814 ENM-based consumer products (coatings, paints and pigments, catalytic additives and cosmetics) commercially available in 32 countries [19]; this number has increased continuously, and currently there are over 3,000 products, according to http://nanodb.dk/. From these products, ENMs are sometimes released in the environment, usually via wastewater; or deposited onto landfills after disposal [9,20,21]. The estimated annual quantity of ENMs released in landfills, water, soil and air was estimated to be 0.3 million metric tons in 2013 [9].

Considering the aforementioned facts, the effects of NMs on aquatic and terrestrial systems have received increasing attention in the past decades [16,20,21,22,23,24,25,26,27,28,29]. NMs of well-defined sizes were shown to actively influence the molecular processes essential for regulating cell functions [30]. Inert NMs can interact with biota via physical pathways such as biological surface coatings [31,32].

Owing to the high proportion of surface versus bulk atoms, even the smallest variations in surface structures may control the fate and reactivity of NMs in air, water, soil, or biota. The tendency for aggregation controlled by the surface charge, size and shape, can reveal new characteristics compared to individual NMs, and can be another important factor to consider in the impact upon the environment [1]. The continuous evolution of analytical techniques has made it possible to put into context the immediate and long-term impact of ENMs on environmental and human health at the local, regional and global levels [9,20,33].

Biological systems have been continuously exposed to and affected by natural NMs [1]. However, in the past century, this exposure increased due to anthropogenic sources (incidental and engineered materials) [34]. The possible mechanisms by which NMs could affect humans were already very well summarized more than 10 years ago [34], and the authors underlined the importance of appropriate risk assessments using a multidisciplinary approach. The hazardous effects of ENMs present in consumer products are under continuous evaluation. However, their continuous evolution in terms of getting combined with other ENMs, getting coated with organic/inorganic layers to enhance their properties or improve their biocompatibility, may pose new health challenges in the future, and regulatory policies will need to keep up with this fast-developing field [35].

## 3. Plant Exposure to ENMs and Toxicity Mechanisms

**Figure 1:**
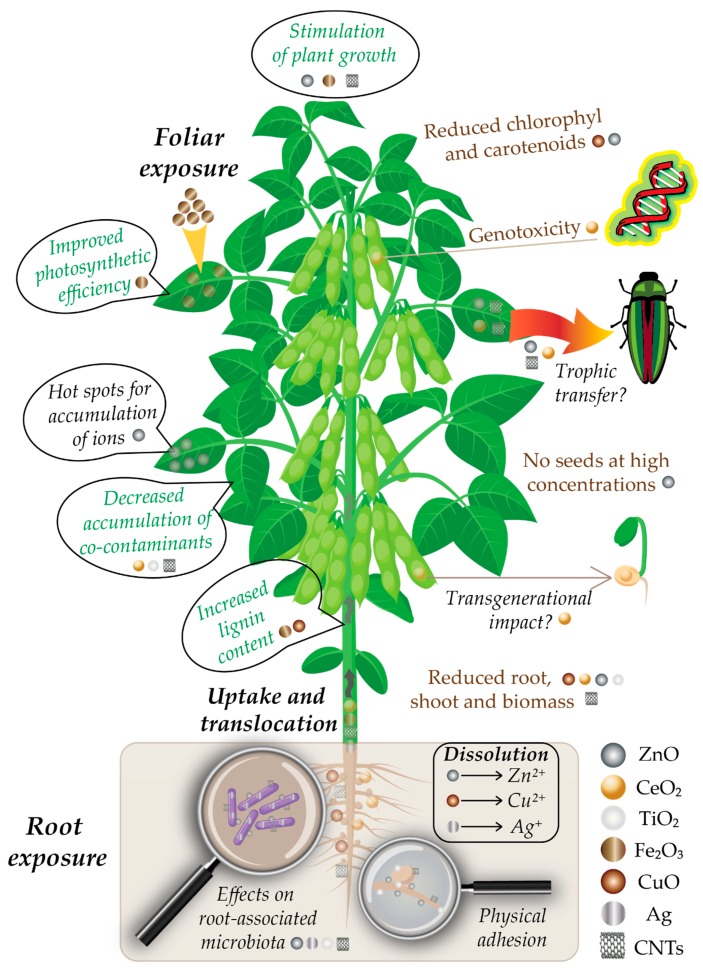
Schematic representation of engineered nanomaterials (ENMs) effects on soybean plants. Positive effects are depicted in green, negative ones in brown, and future research needs are followed by question marks.

Since plants are the dominant kingdom on Earth [11], they have the highest probability to be exposed to different ENMs reaching the environment. The three major sources of terrestrial plant exposure to nanomaterials are the air, water and soil, with the highest amounts of environmental accumulation in landfills and soils [9,20,26,36,37]. Wastewater effluents are a key aspect here, since wastewater sludge is often used as agricultural fertilizer, ending up being spread onto cultivated agricultural fields [1,38,39]. From the soil, NPs are taken up in the plant roots and can bioaccumulate in the plant cells and tissues [10,12,13,16,26,27,34,40,41]. Soil properties (pH, ionic strength, organic matter, composition) can influence the interaction between terrestrial plants and ENMs [42]. Additionally, root-associated microorganisms play an important role in the dissolution and transformation of ENMs, which further influences the uptake of ENMs by plant cells [13,43]. A schematic representation of the uptake and effects of ENMs on soybean plants is given in Figure 1.

From plants, ENMs can enter the food chain via trophic transfer [44], or they can affect the second generation of plants [45] or other living organisms [33,46]. Most importantly, through the food chain, ENMs can also reach the human body, making it imperative to develop a deep understanding of their interactions with the living organisms, their potential accumulation/biomagnification and toxicity mechanisms [25,28,29,35,46,47,48,49,50,51].

Despite many publications in the field, the underlying mechanisms of NM toxicity have still not yet been fully understood, most likely since there are so many different experimental parameters even regarding the use of a single type of NM. The toxicity of a specific ENM depends on a series of factors such as: NM type, concentration and exposure time, degree of aggregation, size and shape, surface-to-volume ratio, surface functionalization, and crystal structure [30,52,53]. Regarding ENMs interaction with plants, both harmful and beneficial effects on physiological, biochemical and genetic levels have been reported in the past two decades [10,12,13,14,36,43,54,55,56,57,58,59,60,61,62,63].

The mechanisms more likely to influence the toxicity of ENMs in plants are: Internalization and accumulation with overproduction of reactive oxygen species (ROS), release of toxic ions, and biophysicochemical interactions (ENMs may interact dynamically with the biomolecules they meet, leading to the formation of protein coronas, particle wrapping, intracellular uptake and biocatalytic processes; in turn, the biomolecules may induce charge transfer reactions, increasing the dissolution at the NP surface). These interactions are mainly governed by properties of the NPs (size, shape, surface charge and coatings) [29,64].

An important mechanism of nanotoxicity is the overproduction of ROS [65,66,67], which results in oxidative stress, lipid peroxidation, protein and DNA damage in plants [21,23,60]. In normal growth conditions, plants actively produce ROS in chloroplasts, mitochondria and peroxisomes, predominantly as signaling molecules for abiotic stress responses and pathogenic defense, among others [65]; and then counterbalance this oxidative stress by deploying their effective ROS scavenging antioxidant machinery (enzymatic and nonenzymatic) [66,67]. The presence of foreign ENMs can however overstimulate ROS production [21,23,60], with negative effects on plant growth and metabolism [57,68].

Oxidative stress is a common mechanism of phytotoxicity documented over a wide range of reactive ENMs with variable physicochemical properties (size, composition, surface chemistry (defects, charge), energy band gap and ionic dissolution levels) [57,69].

Therefore, the presence of ENMs inside cells can impact the cellular redox balance, leading to an overproduction of ROS, depending on their physicochemical properties [69]. For metal oxide NPs, the most frequently-detected indicators of ROS-induced phytotoxicity are the increased production of H_2_O_2_ and malondialdehyde, and altered activities of enzymes such as catalase, superoxide dismutase and ascorbate peroxidase [57].

The level of oxidative stress is concentration-dependent: High-dose ENMs exposure does result in ROS overproduction and therefore cytotoxicity; low-dose exposure (e.g., ZnO < 50 mg/kg of soil) may lead to non-toxic modulation of redox signaling, which may influence disease initiation and progression [69,70]. Oxidative stress can also be a consequence of the ENM-mediated redox state modulation of thiol sulfur switches, which normally function in cell signaling and physiological regulation [71,72].

Some metal and metal-based NPs can dissolve in biologic environments [38], therefore releasing metallic ions which can be up-taken by the plants at greater rates and have higher reactivities than their NP parent. Ag NPs are a very good example, with numerous studies assessing their environmental (bio)transformation and biological effects on plants [73]. Dissolution of Ag NPs is more likely to occur for the smaller particle size (decreased surface area) [74]. ZnO and CuO are other very good examples which cause serious oxidative damage through ionic dissolution [75,76]. For ZnO, toxicity was shown to be a combination of ROS overproduction, NP and ionic toxicity [77,78,79], although ionic toxicity was shown to be more pronounced than the others [79].

For inert NPs (those not releasing toxic metal ions, e.g., organic NMs) during the aquatic life cycle, adsorption at biological surfaces is proposed as a toxicity-related mechanism [10,80]. These effects are evidently mostly size- and charge-dependent [30,53]. Some metal oxides NPs are also known to be relatively inert in biological media (e.g., TiO_2_, Fe_3_O_4_) [81,82], and the formation of a biomolecule corona around them can have a tremendous effect upon their subsequent behavior, including uptake rates [31,83]. In this case, the medium pH, inorganic matter and the occurrence and diversity of the natural organic matter in the soil can have both stabilizing and destabilizing effects on the ENMs [83]. Accumulation of TiO_2_ was shown to have detrimental effects by reducing the hydraulic conductivity of the cell wall of maize seedlings, leading to reduced transpiration and leaf growth [84]. Fe_3_O_4_ NPs adsorbed on the root surface of pumpkin plants were thought to cause local instability of the cell wall [85].

## 4. Soybean Interactions with ENMs

Soya bean has proven to be a perfect model for metal accumulation studies due to high biomass production and ease of cultivation. Numerous data have been published in the past decade about the effect of various ENMs on soybean (Table 1), mainly focusing on ZnO [86,87,88,89,90,91,92,93,94], CeO_2_ [86,87,88,89,90,91,92,95,96,97,98,99,100,101,102], TiO_2_ [95,103,104,105], Fe_2_O_3_ [106,107,108,109,110], Fe_3_O_4_ [104], CuO [111], Cr_2_O_3_ [112], Ag [113,114,115,116] and carbon-based materials [117,118,119,120,121,122,123], and the results are summarized in the following sections.

### 4.1. ZnO and CeO_2_ NPs

The bioaccumulation and biotransformation of ZnO and CeO_2_ NPs in soybean grown in hydroponic, greenhouse or NP-impacted soil conditions, have been investigated extensively over the past 10 years [86,87,88,89,90,91,92]. López-Moreno et al. [86] used X-ray absorption spectroscopy to show for the first time that cubical CeO_2_ NPs (7 nm) were stored in the roots of the plants grown hydroponically in the nanoform, while hexagonal ZnO NPs (8 nm) could not be determined in the roots except as the Zn (II) ionic form, demonstrating a material-dependent effect on soybean plants. It was already shown previously that dissolution plays an important role in ZnO-induced plant cytotoxicity, while the antioxidant properties of CeO_2_ may protect cells from oxidant injury [128]. ZnO NPs were shown to dissolve in aqueous conditions to form hydrated Zn^2+^ cations [10,57,124]. Germination of seeds was not significantly affected by either of the NPs, except for CeO_2_ at 2 g/L, but CeO_2_ was shown to significantly increase root elongation at all tested concentrations (0.5–4 g/L). ZnO-treated seedlings also showed an increased root length at lower concentrations, with a maximum length at 0.5 g/L; however, root length decreased compared to control at higher concentrations, and reached a minimum at 4 g/L [86]. CeO_2_ was shown to exhibit genotoxic effects (DNA damage) on the soybean plants, but only at the highest concentrations tested (2 and 4 g/L), which strongly exceed realistic environmental concentrations, which, in the European Union (EU) for example, have been estimated to be in the ng/L range in surface water and µg/kg in soils [125].

A few years later, the first study of a soybean grown to maturity in NP-impacted soil showed that plant growth (root and shoot length, number of leaves) was negatively affected even at low CeO_2_ NPs (8 nm) concentrations (0.1 g/kg soil), but slightly stimulated by ZnO NPs (8 nm) at similar concentrations in the soil (0.05–0.1 g/kg) [87]. CeO_2_, as assessed via energy dispersive X-ray spectroscopy (EDX) and dark-field scanning transmission electron microscopy (TEM), accumulated mainly in the roots and nodules (very little aboveground translocation) with similar rates to those from the previous hydroponic study [86].

The accumulated quantities of Ce in the roots were 400 times higher at 1 g/kg compared to 0.1 g/kg of soil, and this accumulation almost shut down the N_2_ fixation in the roots. Unlike Ce, Zn accumulated in a dose-dependent fashion in the stem, leaf and pod tissues (visible by TEM). The concentration in the leaves, determined with ICP-OES or ICP-MS, was three times higher than that in the stem, suggesting that leaves act as “hot spots” for Zn bioaccumulation [87]. Since the bioaccumulation results were close to those from a previous study with Zn salts [126], the authors suggested that most of Zn present in plant cells may have dissolved from the NPs [87].

In a subsequent study, Hernandez-Viezcas et al. [88] showed via X-ray absorption spectroscopy that CeO_2_ NPs (8 nm) remained mainly as particles in the root and nodules, while for ZnO (10 nm), the presence of NPs could not be confirmed; various Zn complexes (resembling Zn citrate or nitrate) were identified within the stem, leaves and beans, but none as NPs [88]. This study also showed that a fraction of CeO_2_ NPs and Zn^2+^ ions reached the reproductive/edible portion of the soybean plant, suggesting that CeO_2_ NPs can potentially reach the food chain and the next plant generation [88]. The transgenerational impact of CeO_2_ NPs was shown on the growth and development of tomato plants, where the second generation seedlings from parents treated with CeO_2_ NPs had smaller biomass, water transpiration and slightly higher ROS content [45].

Next, Peralta-Videa et al. [89] have shown that CeO_2_ (8 nm) and ZnO NPs (10 nm) had different effects on the accumulation of nutrients in soybean plants grown in NP-impacted farm soil, therefore altering the nutritional value of the plants. CeO_2_ exposure (0.1–1 g/kg) interfered mainly with the uptake of the chemical elements responsible for nitrogen metabolism and photosynthesis: The levels of Ca, Mg, K and Na decreased significantly, but P and Cu increased in pods. ZnO exposure (0.05–0.5 g/kg) impacted the most the accumulation of essential elements, increasing Zn, Mn and Cu in pods, and Mo in nodules, but decreasing Fe accumulation in leaves [89]. As in the previous study [88], Zn was significantly present in all analyzed soybean organs, including pods.

In a similar study, Ge et al. [90] investigated the effect of CeO_2_ (nanorods; 67×8 nm^2^) and ZnO (spherical NPs; 24 nm) on the bacterial communities present in the soil, and found that ZnO significantly impacted bacterial communities in a dose-dependent manner both in unplanted and planted soils, increasing *Rhizobium* and *Sphingomonas*, but decreasing *Ensifer*, *Rhodospirillaceae*, *Clostridium* and *Azotobacter*. The presence of soybean plants had different effects on soil bacterial communities affected by ZnO. In planted soils, soybean decreased the impact of ZnO NPs on the bacterial communities as compared to the unplanted soils (50% fewer sensitive bacterial operational taxonomic units), most probably by immobilizing the NPs via the root exudates, or by absorbing Zn ions via the roots, and therefore limiting the availability of Zn to soil bacteria [90]. Of note, ZnO was previously shown to have excellent antimicrobial properties [127]. Contrarily, CeO_2_ had little effect on the bacterial communities, except for the lowest concentration tested (0.1 g/kg). In this case, CeO_2_ had a shifting effect on bacterial communities only in planted soils, which was attributed to the previously-reported detrimental effect on the plant root, where plants exposed to this concentration had significantly shorter roots than CeO_2_ NP-free controls [87]. The similarities between the two studies were also confirmed at higher concentrations, where the effects of CeO_2_ were minimal both on the microbial communities and the plant itself [87,90].

While most of the aforementioned studies evaluated the effect on plant growth, a later study from the same groups aimed to evaluate the effects of CeO_2_ (8 nm) and ZnO NPs (10 nm) on the metabolism of the soybean in terms of chlorophyll production, protein content of seeds, ROS, lipid peroxidation and genotoxicity [91]. Both NPs had negative effects, but the most visible damage was seen for CeO_2_ NP exposure: An increased ROS and lipid peroxidation and decreased total chlorophyll concentrations were more pronounced with medium (0.5 g/kg soil) and high (1.0 g/kg soil) doses. These effects correlated well with leaf damage, lower pod and stem production, and a lower root nodule nitrogen fixation potential. Plant growth, yield and N_2_ fixation potential were not affected by ZnO, but moderate oxidative stress, with slightly lower chlorophyll concentrations (attributable to Zn complexes) was observed in a dose-dependent manner. ROS levels were comparable to control, while some genotoxicity appeared in one plant exposed at the highest concentration tested of ZnO (0.5 g/kg soil), mainly attributed to ionic Zn oxidative phytotoxicity [91].

The mild effects of ZnO on the soybean have been contradicted by the results from another study with plants cultivated in a standard soil microcosm (57–65 days), which has shown that ZnO NPs (<50 nm) at 0.05 and 0.5 g/kg soil affected significantly developmental stages and reproduction of the soybean plants: Roots were smaller, and no seeds were formed at the highest concentration (0.5 g/kg) [92]. However, compared to the previous studies, the ZnO NPs in this study were larger by an order of magnitude, and their size might have influenced their biological effects, since the NPs properties are highly size-dependent [30,53]. While smaller ZnO particles were shown to dissolve in the soil and enter plant cells as Zn^2+^ cations, larger size particles could enter the cell compartments as NPs and have biologically different effects [128]. Higher dissolution rates would be expected for smaller sizes due to an increase in the surface specific area [77].

In another study, exposure to CeO_2_ NPs (25 nm) at 0.25–1 mg/mL did not significantly affect soybean seed germination or early development root length [95]. In plants grown hydroponically, Dan et al. [96] found dissolved Ce for the first time in plant seedling shoots exposed to CeO_2_ NPs. The impact on soybean was shown to be affected by the soil moisture content, with positive effects above 70% humidity [97,98]. In terms of interactions with other co-contaminants, CeO_2_ and Cd were found to interact significantly, affecting each other’s accumulation, and consequently, their biological effects [99,100]. As expected, the effects were concentration-dependent [101], and soil sterilization was shown to have a significant impact on the effect of NPs, most probably due to the alterations of the soil microbiota [102].

In general, the above studies show that ZnO may have a positive effect at low concentrations (50 mg/kg) on the seed germination and early development (increased root length), with a promising potential against plant disease due to its antimicrobial properties. This observation was confirmed for many other terrestrial plants, as comprehensively summarized recently [129]. However, the effects are concentration-dependent, with negative outcomes in the high acute ranges (>500 mg/kg), where an overproduction of ROS often leads to cytotoxicity, unless there is a deficiency of Zn in the growing media [129].

Most studies considered that the dissolution of ZnO to Zn ions plays the main role in the effects of these NPs on the soybean plants. As expected, dissolution was particle size-dependent, with smaller NPs being more susceptible to being solubilized. Proteins and organic substances can also increase the dissolution rates of ZnO through ligand-enhanced dissolution [128]. A very recent study with multiple plant crops has shown that soil pH and plant species were key factors affecting ZnO uptake and phytotoxicity [130]. Since many studies concur that ZnO toxicity can be mainly explained by the release of toxic ions, an important aspect needing further clarification in the nearest future is whether we should evaluate metal-containing NPs prone to dissolution using the existing legislation for heavy metals, or possibly include a correction for higher availability in agricultural soils.

In terms of bioaccumulation in soybean plants, ZnO was distributed in all soybean plant organs, in a concentration-dependent manner. The presence of Zn in the edible parts of the exposed plants rises questions on possible transgenerational effects, trophic transfer and biomagnification [44]. So far, literature data on ZnO trophic transfer and biomagnification are limited [131]. One example assessing the trophic transfer of ZnO NPs (30±17 nm) from daphnids to zebrafish found a tenfold higher bioaccumulation compared to water exposure in other studies [132]. No studies have been found related to ZnO NP trophic transfer from plants to animals. Therefore, further research is needed to elucidate these aspects, especially since trophic transfer is a possible pathway for human exposure to ENMs [133].

Contrastingly to ZnO, CeO_2_ was shown to be mainly present in the below ground parts of the soybean plant (root and nodules), and not to be prone to biotransformation. CeO_2_ NPs are stable and insoluble in biological systems, and therefore they could be found in the roots of soybean as NPs. Both positive and negative effects were documented, CeO_2_ acting either as an antioxidant or as a ROS producer in soybean plants. In *Brassica rapa*, the responses to CeO_2_ exposure were also shown to vary with particle size and plant growth stages [134]. One study suggested possible transgenerational effects of CeO_2_, as a fraction of NPs was found in the seeds of the exposed plants [85].

Trophic transfer studies with CeO_2_ NPs have shown that transfer and biomagnification of Ce occurs within the studied food chains [135,136,137].

### 4.2. TiO_2_ NPs

So far, the effects of TiO_2_ NPs on soybean are not as pronounced as those seen with CeO_2_ or ZnO [95,103,104,105]. TiO_2_ had only marginal effects on seed germination [95], plant growth, nutrient content and the composition of bacterial communities within the rhizosphere [103,104]. However, in terms of co-contaminants, TiO_2_ was shown to restrict Cd-induced plant toxicity (reduced plant growth and biomass, pigment and protein content) by increasing the photosynthetic rate and growth parameters of the plants [105]. Reducing the effect of dangerous co-contaminants may potentially be used for bioremediation of soil pollution [61,138]. Again, the TiO_2_ NP diameter can play a significant role in uptake, accumulation and effects, as shown for many other NPs [30]. An experiment performed by Larue et al. [82] in wheat (*Triticum aestivum*) has shown that TiO_2_ NPs < 36 nm accumulated in roots and were distributed in the whole plant tissues, NPs 36–140 nm accumulated in the root parenchyma, but did not translocate to the shoots, and NPs > 140 nm did not accumulate in the roots. In all studies with soybean, the NP diameters were <100 nm, and were found in the plant roots, but with marginal effects on plant growth.

Many other studies in other plants, summarized in several reviews [10,13,57,61,62,139,140,141], have shown both positive (antimicrobial effect that can promote growth) and negative effects (oxidative stress, cytotoxicity, genotoxicity, reduced germination rates, reduced root and shoot growth and development). Compared to ZnO, TiO_2_ translocation in plant tissues happened without the biotransformation or dissolution of NPs [81,82]. In a study with cucumber, Servin et al. showed that TiO_2_ NPs could enter food chains by translocation from root to fruit without biotransformation [142]. A similar result was obtained with tomatoes, when the plants were exposed at higher concentration of NPs (i.e., 130 mg/kg) [143]. In a recent study evaluating trophic transfer, Chen et al. has shown that biomagnification factors of daphnia fed with algae exposed to TiO_2_ (1–10 mg/L) were between 5.7 and 122 [144].

A very recent review has summarized the possible mechanisms of the toxicity of TiO_2_ to living organisms into three main categories [145]: (i) ROS via generation of photo-induced electron-hole pairs; (ii) electrostatic attachment (of positively-charged TiO_2_) to cell walls—causing wall damage and lipid peroxidation; and (iii) attachment to internal organelles (e.g., mitochondria) and macromolecules, post internalization. In our opinion, these three aspects may be the main mechanisms that can be attributed to most inert NMs up taken by living organisms (including plants).

### 4.3. Iron Oxides (Fe_2_O_3_ and Fe_3_O_4_) NPs

Fe_2_O_3_ NPs were shown to have mainly positive effects on soybean growth in terms of biomass increase [106], concentration-dependent protein, lipid, fatty acids and minerals [107], root elongation and shoot weight [108], chlorophyll content [107,109] and an increased lignin content of roots [110]. In the last-mentioned study, however, the increased lignin content in roots caused a detrimental effect on plant growth [110]. Fe_2_O_3_ NPs were applied via foliar spraying [106,107,108], medium [109,110] and soil amendment [108]. Alidoust and Isoda [108] showed that a foliar application of Fe_2_O_3_ (6 nm; 0.05–2 g/L) gave better results in terms of photosynthesis efficiency than soil amendment. Additionally, increased stem and root growth and overall yield, were observed, and the effects were more pronounced than those observed with Fe in the ionic form (salts) [108]. Positive effects of iron oxide NPs were also seen in other plants [146]. The surface charge of the NPs of Fe_3_O_4_ had an important effect: Contrarily to the positively-charged NPs, negatively-charged NPs increased leaf P content, translocation of Fe to leaf tissues, and decreased the root colonization of rhizobia [104]. This suggests that the negatively-charged could increase biocompatibility and uptake of Fe_3_O_4_ NMs by plants [147].

### 4.4. CuO NPs

A similar effect in terms of increased lignin content in the root and a decrease in root growth as for Fe_2_O_3_ [110] was seen with CuO NPs (50 nm) [111]. The authors explained the effect by the increased activity levels of both cationic and anionic peroxidases [148]. In other studies in wheat, CuO was shown to be more toxic than ZnO, and the toxicity was higher for smaller particle sizes [75,76]. This is not surprising, as it was shown that the release of ions through dissolution is the main mechanism by which CuO NPs cause oxidative stress, and that dissolution is more pronounced when the NPs are smaller [55]. The authors also showed that environmental factors from both the cultivation medium and the plants can influence the aggregation or dissolution of NPs, and therefore altering their effects on the plants [75,76]. In another study, CuO NPs were shown to induce DNA damage in agricultural and grassland plants [149]. A recent review summarized the interaction between CuO NPs and cultivated crop plants, and showed that the main toxic effects are caused by: (i) inhibition of seed germination (barley, cilantro, cucumber, lettuce, rice); (ii) decreases in root and shoot sizes (alfalfa, carrot, soybean, wheat, cotton); (iii) reduction of photosynthesis and respiration (maize, tomato); and (iv) morphological and enzymatic changes (rice, soybean, wheat) [55].

### 4.5. Cr_2_O_3_ NPs

A study with Cr_2_O_3_ NPs (30–50 nm; 0.01–0.5 g/L) and soybean aimed to confirm if these NPs can be absorbed and translocated by plants; whether they may affect the chloroplasts and chlorophyll production [112]. The authors have shown that Cr_2_O_3_ NPs caused root swelling and decreased plant growth by lowering the activity of the photosynthetic enzymes (malate dehydrogenase and Ribulose-1,5-bisphosphate carboxylase/oxygenase—Rubisco), altering the substructure of chloroplasts, and reducing the formation and activity of chlorophyll, therefore inducing irreversible damage to the soybean plants.

### 4.6. Ag NPs

Ag NPs interaction with soybean was not shown to have a negative impact on germination and growth [115]. Ag reduced flooding stress by shifting from fermentation to normal cellular processes [114]; and reduced dichlorodiphenyldichloroethylene’s (DDE; a co-contaminant) negative effects by lowering its accumulation rate [113]. However, in a very recent study with transgenic and non-transgenic soybean plants, Galazzi et al. [116] have shown that biomass production was reduced in the presence of Ag NPs, suggesting the presence of oxidative stress in the plants. Many studies in other plants have reflected both the positive and negative effects of Ag NPs on seed germination, and some were summarized in a relatively recent review [62]. Another very recent review has summarized the recent advances related to Ag NPs (synthesis, transformation in the environment and toxicity) [73]. In biological environments, Ag NPs can either dissolve, aggregate or adsorb at the surfaces, but also interact with other molecules (organic and inorganic), depending upon their size, medium pH and surface characteristics [73]. Aggregation induced by extracellular metal-binding polypeptides and proteins plays an important role in limiting NPs dispersion in natural environments [150]. It is well-known that Ag NPs can attach to sulfur proteins from bacterial membranes, enter bacterial cells and then attack the respiratory chain and cell division, finally leading to cell death [151,152,153]. This antimicrobial effect is another important mechanism that can possibly influence the plant root microbiota, which has a crucial role in the acquisition of nutrients from the soil [43].

### 4.7. Carbon Nanotubes and Fullerenes

Studies performed on soybean plant exposure to multi-walled carbon nanotubes (MWCNTs) and C_60_ fullerenes show both positive and negative influences. For example, Begum et al. found no signs of toxicity caused by MWCNTs exposure of soybean plant in hydroponic cultures [117], but observed decreases in the roots, shoots and biomass, as well as cell death and membrane damage in lettuce, red spinach, cucumber and rice—suggesting that the phytotoxicity effects are species-related.

Positive effects on the germination rate and growth of soybean seedlings upon MWCNTs exposure was documented by Lahiani et al. [118], with no observed negative effects on plant development. The study also reports an increased expression of genes encoding several types of water channel proteins. In a later hydroponic study, the same authors [119] showed that MWCNTs impacted the root growth in the soybean, diminishing both the fresh and dry weight of the roots, while no other negative effects were observed on the evolution of other plant organs. Positive effects observed include phenotypical changes and a stimulation of photosynthesis. Using Raman spectroscopy, MWCNTs were detected in roots, stem, leaves and seeds. The presence of CNTs in leaves and seeds raises again the issue of possible trophic transfer. Some studies published so far argue that the overall risk of trophic transfer is low for CNTs [154].

A relatively recent study found several inhibitory effects caused by MWCNTs exposure [120]. The total root length of seedlings was decreased by 29%, and the formation of lateral roots and the shoot growth were also inhibited. Oxidative stress was induced; some antioxidants, e.g., proline and micronutrient supplementation (Zn, Fe, Mn) could revert the growth inhibition caused by exposure to MWCNTs. Zhai et al. [121] found that differently-charged MWCNTs (positive, neutral, negative) were accumulated by the soybean plant (roots, stems, leaves); the inhibition of transpiration and growth of dry weight biomass were reported for all types of nanotubes. They were transported from roots to specific locations in leaves, depending upon their charge and size. Although MWCNTs inhibited soybean growth, the same study showed a stimulation of maize growth, proving once again that the effects are dependent on the plant species [121].

In another study, exposure to either MWCNTs or C_60_-induced phytotoxicity in soil-cultivated plats [122]. MWCNTs reduced the biomass by 19.2–26.9% and the net growth by 29.8–31.9%, and C_60_ fullerenes reduced the biomass by 25.0–40.4% and the net growth by 27.7–42.6%. MWCNTs decreased the accumulation of pesticides chlordane and DDx (DDT and metabolites), and C_60_ increased chlordane accumulation. Exposure to C_60_ fullerenes resulted in oxidative stress, with cell membrane damage of roots and shoots. C_60_ lead to increase in the bioaccumulation of DDE, a persistent pollutant [123]. DDE shoot content was decreased by 48.0%, but the root and whole plant levels were increased by 47.7% and 45.7%, raising questions about potential risks on enhancing accumulation levels of soil contaminants.

The somehow contradicting results regarding the phytotoxicity of C-based materials on the same plant species can be due to the fact that in most of the above-mentioned studies, the toxicity was assessed on a macro level (germination and growth), and not in depth on a molecular level (cell morphology, metabolite production, genotoxicity). Sometimes, the toxic effects fail to influence the overall plant development (biomass, growth, germination), but can affect the plant genotype, metabolite production or can produce oxidative damage [155]. Moreover, C-based ENMs uptake was shown mainly in hydroponic studies, and was less frequently studied in soils. Bioavailability in soils and bioaccumulation in plants may be very different depending on soil type and organic matter content [154]. The strong affinity of CNTs for organic macromolecules in soils is well known [156].

## 5. Plant Microbiota and the Influence of ENMs

Understanding the plant-bacterial interaction mechanisms is crucial to elucidate the environmental changes impact on agriculture and for biotechnological applications to improve production in unfavorable environments. Plant-associated bacteria (microbiota) have an important ecological role in plant development, by promoting plant growth and productivity, and providing resistance to disease [157,158,159].

In terms of promoting plant growth, phosphorus (P) uptake, one of the most important processes in plant nutrition, is increased by phosphate-solubilizing bacteria, which use inorganic phosphates applied to the soil as fertilizers [160,161]. Nitrogen fixation is another important process for plants where bacteria can have a crucial role [161,162,163,164]. N and P are key nutrients that limit agricultural sustainability around the globe [165]. In terms of protection against disease, bacteria can act as biocontrol agents against pathogenic bacteria and fungi, by competing effectively for colonization sites or nutrients, and by producing fungistatic compounds and bacterial allelochemicals [159,166].

Thus, the plant microbiota is involved in diverse biochemical mechanisms, as demonstrated by various studies about the bacterial influence on plant growth and health [43]. When present in soil, NPs can influence the plant microbiota, and especially the microbes associated with the root, with significant effects on plant growth. For soybean, this is the case for ZnO [87,90,92], CeO_2_ [87] and TiO_2_ [104]. Some of these studies show that the effects could be selective, meaning that only some bacterial groups could be affected by certain NPs [167]. For example, Priester et al. [87] found that ZnO had relatively little effect on nitrogen-fixation bacteria, but CeO_2_ had practically shut down nitrogen fixation at high concentrations. NPs’ influence on plant microbiota could be mainly a result of their antimicrobial properties, which are dependent upon size, surface reactivity and charge [127,151,152,168]. Soil type is also an important factor affecting the interaction between NPs and microbiota; organic matter present in the soil can interact with NPs and reduce their toxicity [169,170].

When investigating CNTs’ effects on root-associated microorganisms, the diversity of the soil microbiota was not influenced significantly in some cases [171,172]. In others, the effects were concentration-dependent [173], and differences were seen between functionalized and non-functionalized CNTs [172,173]. In other plants (e.g., tomato), the effects of CNTs were beneficial in terms of the amounts of flowers and fruits [171].

## 6. Conclusions and Perspectives

The current review paper summarized the data published in recent years (past two decades) about the impact of ENMs on soybean, a very important crop in terms of protein and oil production for human food. The interactions between soybean and ENMs were analyzed in terms of growth and production, influences on metabolism and on the plant-associated microbiota.

Several conclusions and outlooks can be drawn from the analyzed data:

(i) The interaction effects between edible plants (including soybean) and ENMs are material-dependent, in terms of NM type (organic vs inorganic), size, shape, surface charge, concentration, solubility and aggregation properties. Several toxicity mechanisms were identified when ENMs had a negative effect on plants: Overproduction of ROS and oxidative stress via uptake and accumulation in plant organs (especially in the roots), release of toxic ions (which can travel and accumulate faster than the parent NM), physical adhesion to biologic surfaces (e.g., protein corona around NPs) and influence on the root-associated microbiota (e.g., selective antimicrobial properties). Oxidative stress is ENM concentration-dependent, with high-dose exposures generating visible damage to the plant, while low-dose exposure (e.g., <50 mg/kg of soil) may seem non-toxic, but with possible effects on the modulation of redox signaling inside cells [69]. Additionally, the ENM effect can vary depending on the application method, e.g., foliar spraying vs soil amendment.

(ii) The phytotoxicity of ENMs are species-related: The same ENM can have positive effects on one plant, be neutral for another, and impede growth and metabolism for a third. However, ENM uptake is common, and the documented presence of NMs in different plant organs and cells highlights the importance of detailed risk assessments of ENM-contaminated plants moving into the food chain. Moreover, the presence of ENMs in the edible parts of the plant raises questions on the possible risk of trophic transfer and biomagnification in animals and humans. Research studies tackling these aspects are scarce, and more research data are needed to clarify these important aspects, that may affect human health.

(iii) Many of the cited studies use ENM concentrations well above the actual levels in the soil and air (estimated in real-life conditions) and the toxic effects would need to be confirmed by studies with plants cultivated in real-life settings (i.e., in soils containing levels of ENMs close to reality). Moreover, more studies are needed related to long-term exposure, and the trans-generational impact of ENM accumulation in the seeds of the parent plant. Performing reliable bioaccumulation measurements will also be important in the future to enable new insights into the interaction mechanisms between plants and NMs of different types, shapes and sizes. New analytical methods, such as single-particle, inductively-coupled plasma mass spectroscopy, show promise to shed light on the size distribution of NMs in biological samples [174].

(iv) Soil represents the main pathway for ENM exposure to terrestrial plants, since the application of ENMs in the agricultural sector (i.e., herbicides, pesticides and fertilizers) has increased constantly over the past few decades. Soil type and composition are important factors in modeling the ENM nano-bio-interactions with plants (e.g., organic matter can bind to ENMs and decrease their transport in soil, and therefore possible toxicity). Future research data should also clarify the impact of surface-coated ENMs on plants, and consequently, on human health.

Particularly for soybean, the positive effects of ENMs in terms of germination and growth have been witnessed with the application of low concentrations of ZnO in soil (50 mg/kg), Fe oxides (both in soil and via foliar application), and sometimes with CeO_2_, Ag NPs and CNTs (under specific conditions). TiO_2_ was mainly neutral, while CuO and Cr_2_O_3_ exposures were detrimental for plant development. Many of the studies were however limited to the plant juvenile developmental stages (germination and seedlings), which limits the analysis of possible effects to plant organs still in development. There were several studies that evaluated the effect of some ENMs throughout the full life cycle of the plants, but more data are needed for a deeper understanding of possible effects. Additionally, future studies should clarify transgenerational effects and possible trophic transfer risks.

To summarize, we are at a stage where we started to be aware of the interconnection between the production and transport of NMs (natural, incidental and engineered), and their impact on our environment and life (plants, animals and humans). Therefore, multidisciplinary comprehensive risk-assessment studies should be carried out prior to the mass productions of potential hazardous materials, which may be otherwise innovative for energy and industrial purposes (e.g., the downside of plastic bioaccumulation and biotransformation in the environment). Moreover, legislation at local, national and international levels needs to keep up with the rapid evolution of the nanotechnology products and technologies.

## Figures and Tables

**Table 1:** Soybean interaction with various ENMs.

NM	Form; Size	Conc.	Growth Conditions	Effects	Ref.
ZnO	NPs hexagonal; 8 nm	0–4 g/L	Germination tests	ZnO NPs dissolved as Zn^2+^ had no effect on germination.	[86]
	NPs powder in soil; ~10 nm	0.5–5 g/kg soil	Garden pots with 2.4 kg of soil	Zn bioaccumulated in all tissues and especially in the leaves. ZnO slightly stimulated plant growth.	[87]
	NPs powder in soil; ~10 nm	0.5 g/kg soil	Same as in [87]	ZnO NPS were dissolved and accumulated in the seeds in a form resembling Zn citrate.	[88]
	NPs powder in soil; ~10 nm	0–0.5 g/kg soil	Same as in [87]	ZnO impacted the accumulation of essential elements (K, Mg). Zn accumulation was significantly increased in all plant organs.	[89]
	NPs powder in soil; ~10 nm	0–0.5 g/kg soil	Same as in [87]	ZnO significantly altered soil microbiota both in unplanted and planted soils; the presence of plants reduced effects on soil bacteria.	[90]
	NPs powder in soil; ~10 nm	0–0.5 g/kg soil	Same as in [87]	ZnO NPs decreased chlorophyll concentrations and had some genotoxic effects at the highest concentration (0.5 g/kg soil).	[91]
	NPs; <50 nm	0–0.5 g/kg soil	Soil (65 d)	ZnO NPs reduced roots and shoots (area and volume). Plants treated with high conc. (0.5 g/kg) had no seeds.	[92]
	NPs; 40–60 nm	0.025–0.5 g/L	Germination tests	ZnO promoted the growth of primary roots and supported the development of first trifoliate leaves earlier than the control.	[93]
	NPs; 41 nm	0–400 ppm	Hoagland medium (21 d)	ZnO NPs reduced chlorophyll and carotenoids, and increased anthocyanin, malondialdehyde, H_2_O_2_ and phenylalanine ammonia-lyase activity.	[94]
CeO_2_	NPs cubic; 7 nm	0–4 g/L	Germination tests	CeO_2_ NPs remained intact in roots and had genotoxic effects.	[86]
	NPs powder in soil; ~8 nm	1–10 g/kg soil	Garden pots with 2.4 kg of soil	CeO_2_ NPs decreased plant growth and yield, stopped nitrogen fixation at high conc.; no effect on seed production.	[87]
	NPs powder in soil; ~8 nm	1 g/kg soil	Same as in [87]	CeO_2_ NPs were in the root nodule including root epidermis and pods. NPs were also shown to potentially transfer to next plant generation via the reproductive organs.	[88]
	NPs powder in soil; ~8 nm	0–1 g/kg soil	Same as in [87]	CeO_2_ interfered with the uptake of elements involved in nitrogen metabolism and photosynthesis (Ca, Mg, P, K, and S).	[89]
	NPs powder in soil; ~8 nm	0–1 g/kg soil	Same as in [87]	CeO_2_ had no effect in unplanted soils; the presence of plants promoted the altering of bacterial communities in planted soils.	[90]
	NPs powder in soil; ~8 nm	0–1 g/kg soil	Same as in [87]	CeO_2_ NPs caused signs of oxidative damage in leaves with consequences to the entire plant.	[91]
	NPs; ~25 nm	0–1 g/kg soil	Germination tests	CeO_2_ NPs did not significantly affect germination and root.	[95]
	NPs; 30–50 nm	7 mg/L	Hydroponic growth	Dissolved Ce was found for the first time in plant seedling shoots exposed to NPs hydroponically.	[96]
	NPs; 10–30 nm	0–0.5 g/kg soil	Greenhouse (3 wk)	At 0.1 g/kg, CeO_2_ NPs stimulated plant growth and photosynthesis (+54%). Photosynthesis rate decreased at 0.5 g/kg (~36%).	[97]
	NPs; 10–30 nm	0.1 g/kg soil	Greenhouse (3 wk)	CeO_2_ NPs was dependent on soil moisture, with positive effects (increased fresh biomass) above 70% moisture content.	[98]
	NPs; ~42 nm	0–0.5 g/kg soil	Greenhouse (30 d)	CeO_2_ NPs enhanced the plant light energy use efficiency by photosystem II. The presence of Cd significantly increased Ce accumulation in plant tissues.	[99]
	NPs; ~42 nm	0.1 g/L (+Cd)	Hydroponic growth	CeO_2_ NPs and Cd interacted significantly, affecting their accumulation: CeO_2_ reduced the translocation of Cd from roots to shoots by 70%; Cd lowered the conc. of Ce in roots by 45% but increased it in shoots by 60%.	[100]
	NPs; 20–200 nm	0–2 g/kg soil	Greenhouse	Root biomass was reduced by 60% and by 81%, while shoot biomass increased by 65% and 92% at 0.5 and 2 g/kg CeO_2_.	[101]
	NPs; ~10 nm	0–0.5 g/kg soil	Greenhouse (27 d)	Initial soil sterilization affected interaction of CeO_2_ NPs with plants and CeO_2_ accumulation. The net photosynthesis rate was higher at 0.1 g/kg but lower at 0.5 g/kg, as compared to the unsterilized soil.	[102]
TiO_2_	NPs; ~25 nm	0–1 g/kg soil	Germination tests	TiO_2_ NPs showed a marginal effect on germination.	[95]
	NPs; <60 nm.	<0.2 g/kg soil	Greenhouse (6 wk)	TiO_2_ was found in roots; no effects on plant growth, nutrient content, or the composition of root-associated microbiota.	[103]
	NPs; 22–25 nm	0–0.2 g/kg soil	Greenhouse (6 wk)	TiO_2_ significantly reduced plant growth.	[104]
	NPs; <100 nm	0.1–0.3 g/kg soil (+Cd)	Plant growth chamber	TiO_2_ NPs restricted Cd-induce toxicity by increasing the photosynthetic rate and growth parameters of the plants.	[105]
Fe_2_O_3_	Sprayed nano-Fe_2_O_3_	0.25–1 g/L	Clay soil (pH 7.6)	Fe_2_O_3_ NPs enhanced pod and grain biomass by 48%.	[106]
				Fe_2_O_3_ NPs at 0.75 g/L enhanced protein (33.8%) and lipid (25.4%) content *vs* control; increased fatty acids, minerals and chlorophyll.	[107]
	NPs; 6 nm. Foliar *vs* soil treatment.	0.05–2 g/L	Wagner pots	Fe_2_O_3_ NPs produced positive effects on root elongation, shoot weight, leaf area, and soil plant analysis development values.	[108]
	Superparamagnetic NPs; 9 nm.	0.2–2 g/L	Hydroponic growth	NPs significantly enhanced the chlorophyll content in subapical leaves, with no trace of toxicity.	[109]
	NPs; <50 nm	0.2–1.5 g/L	Hoagland nutrient solution	Fe_2_O_3_ NPs increased the lignin content of roots and stems, followed by the stiffening of the cell wall and growth inhibition.	[110]
Fe_3_O_4_	NPs; 18 nm	0–0.2 g/kg soil	Greenhouse (6 wk).	Fe_3_O_4_ NPs increased plant growth and leaf C but reduced P content. Negatively charged Fe_3_O_4_ NPs increased leaf P content, and decreased root colonization of rhizobia, *vs* positive NPs.	[104]
CuO	NPs; 50 nm	0–0.5 g/L	Murashige and Skoog medium	CuO NPs increased lignification of root cells via improving root peroxidases activity; and reduced the shoot growth, weight, and total chlorophyll content.	[111]
Cr_2_O_3_	NPs; 50 nm	0.01–0.5 g/L	Suspensions with NPs	Cr_2_O_3_ NPs inhibited plant growth by damaging photosynthesis, destroying the chloroplast thylakoid structure and inhibiting electron acceptors.	[112]
Ag	NPs or bulk; 68–91 nm.	0.5–2 g/L	125 mL jars of vermiculite	Ag NP-exposed plants had 1.9−2.2 x higher Ag content and transport to shoot tissues. Ag altered DDE (a co-contaminant) accumulation and translocation.	[113]
	NPs; 2–80 nm	0.2–20 ppm	Soybean exposed to flooding stress	Ag NPs positively influenced the growth performance of soybeans under flooding stress.	[114]
	NPs; ~60 nm	0.15–0.31*10^12^ NPs/mL	Standard germination tests	Ag NPs did not present any negative effects on the germination and growth.	[115]
	NPs; ~60 nm	50 mg/kg *vs* AgNO_3_	Greenhouse (21 d).	Ag NPs decreased the mass production of non-transgenic plants by 25% by generating oxidative stress.	[116]
C	MWCNTs, od 13 nm, id ∼4 nm, l >1 μm	0–2 g/L	Hydroponics (15 d)	MWCNTs induced very little or no effect on root and shoot growth, cell death, and electrolyte leakage at the seedling stage.	[117]
	MWCNTs, od 15–40 nm; l - several μm	0–200 μg/mL	Agar medium	MWCNTs accelerated seed germination, increased roots, and showed no negative effects on plant development.	[118]
		50 μg/mL	Hydroponics (20 wk)	MWCNTs decreased the roots weight; no influence on shoot reduction or the development of other organs was observed; CNTs enhanced photosynthesis.	[119]
	MWCNTs; od 20–70 nm, id 5–10 nm, l >2 μm	1 g/L	Suspensions with CNTs (36 h)	MWCNTs treatment reduced the total root length by 29%, induced oxidative stress.	[120]
	MWCNTs, d 20−30 nm, l - 0.05−2.0 μm	10–50.0 mg/L	Hydroponics (18 d)	MWCNTs inhibited growth and transpiration; increased dry weight biomass (@20 mg/L); the effect was influenced by the MWCNTs charge.	[121]
	MWCNTs, hd 3.5–3.9 μm (0.5–1 g/L) and 17.7 μm (5 g/L)	0–5 g/kg soil	Soil (28 d)	MWCNTs induced phytotoxicity; reduced biomass (19.2–26.9%), reduced net growth (29.8–31.9%). Co-exposure with contaminants decreased chlordane and DDx accumulation.	[122]
	C_60_ (fullerenes), 1450-1900 nm	0–5 g/kg soil	Soil (28 d)	C_60_ induced phytotoxicity; reduced biomass and net growth by 25.0–40.4% and 27.7–42.6%. Co-exposure with contaminants increased chlordane uptake.	[122]
		40 mg/80 mL	Vermiculite (3 wk)	Fullerene treatment decreased accumulation of p,p′-DDE contaminant in shoots (48%); root and total plant p,p’-DDE increased.	[123]

NPs – nanoparticles; d – day; wk – week; DDE – dichlorodiphenyldichloroethylene; d – diameter; l – length; id/od/hd – internal/outer/hydrodynamic diameter.
